# Supplementary figures and images for: Novel ABCD1 and MTHFSD Variants in Taiwanese Bipolar Disorder: A Genetic Association Study
Source: Medicina (Kaunas). 2025 Mar 11;61(3):486. doi: 10.3390/medicina61030486 (PMC11943623; doi:10.3390/medicina61030486)

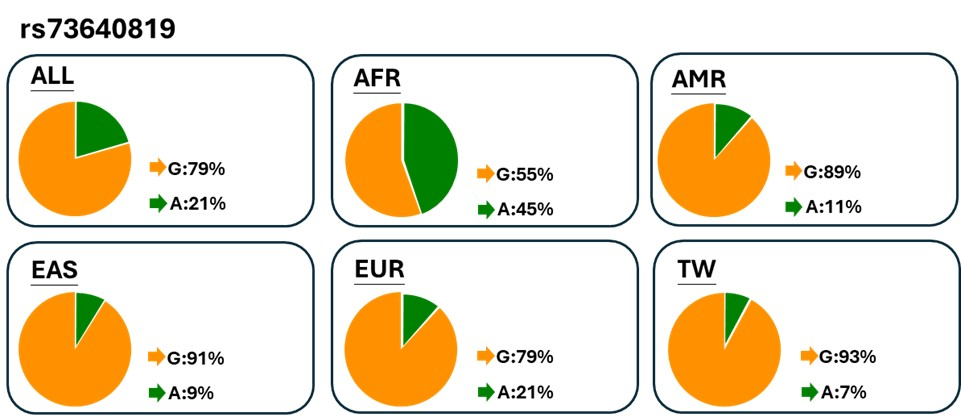

Supplement: Supplementary file 1 [file medicina-61-00486-s001.zip › Supplementary Figure S1.tif]

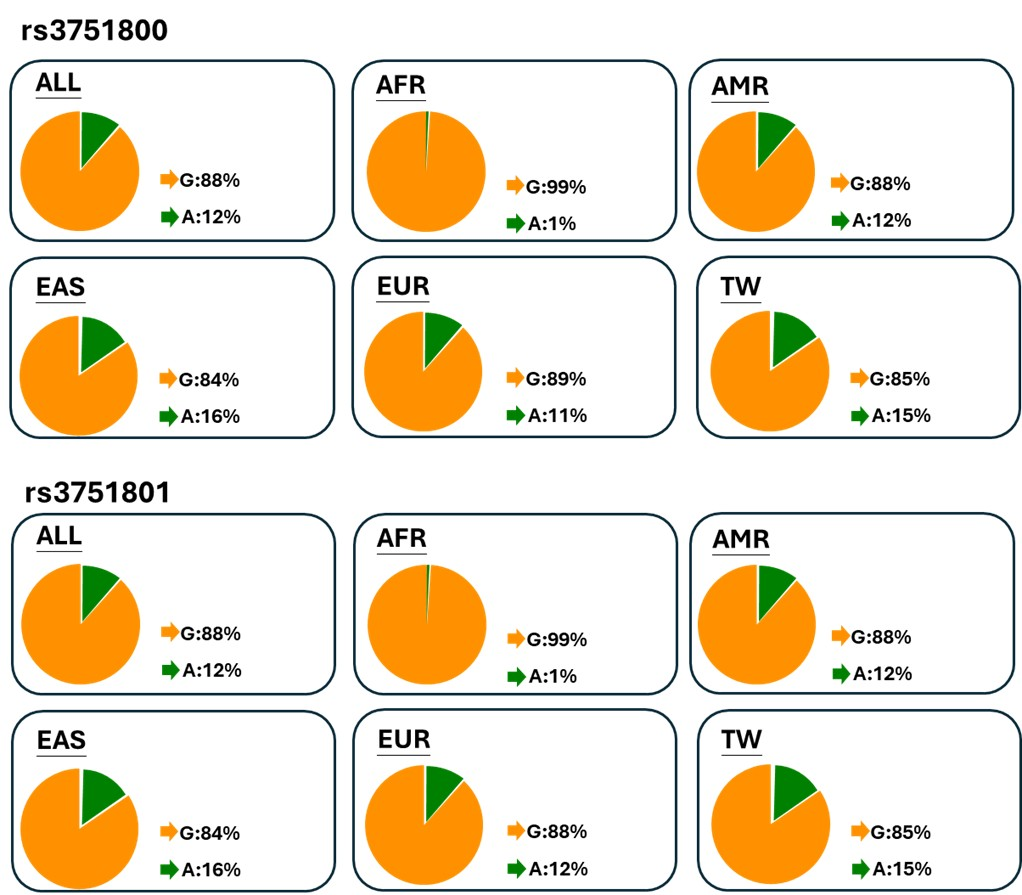

Supplement: Supplementary file 1 [file medicina-61-00486-s001.zip › Supplementary Figure S2.tif]
